# Antagonism of nonaflatoxigenic *Aspergillus flavus* isolated from peanuts against aflatoxigenic *A. flavus* growth and aflatoxin B_1_ production *in vitro*

**DOI:** 10.1002/fsn3.2995

**Published:** 2022-08-24

**Authors:** Mohd Azuar Hamizan Rahman, Jinap Selamat, Nik Iskandar Putra Samsudin, Khozirah Shaari, Norlia Mahror, Joshua Mark John

**Affiliations:** ^1^ Department of Food Science, Faculty of Food Science and Technology Universiti Putra Malaysia Serdang Malaysia; ^2^ Laboratory of Food Safety and Food Integrity, Institute of Tropical Agriculture and Food Security Universiti Putra Malaysia Serdang Malaysia; ^3^ Department of Chemistry, Faculty of Science Universiti Putra Malaysia Serdang Malaysia; ^4^ Natural Medicines and Product Research Laboratory, Institute of Bioscience Universiti Putra Malaysia Serdang Malaysia; ^5^ Food Technology Division, School of Industrial Technology Universiti Sains Malaysia Pulau Pinang Malaysia

**Keywords:** aflatoxigenic, aflatoxin B_1_, *Aspergillus flavus*, biocontrol, colony growth rate, non‐aflatoxigenic

## Abstract

*Aspergillus* section *Flavi* constitutes several species of opportunistic fungi, notable among them are *A. flavus* and *A. parasiticus*, capable of surviving harsh conditions and colonizing a wide range of agricultural products pre‐ and postharvest. Physical and chemical control methods are widely applied in order to mitigate the invasion of *A. flavus* in crops. However, physical control is not suitable for large scale and chemical control often leads to environmental pollution, whereas biological control offers a safer, environmentally friendly, and economical alternative. The present study aimed to investigate the antagonism of several non‐aflatoxigenic *A. flavus* strains against the aflatoxigenic ones *in vitro* (semisynthetic peanut growth medium; MPA) in terms of colony growth rate and AFB_1_ inhibition. Different peanut concentrations were used to obtain the optimum peanut concentration in the formulated growth medium. A dual culture assay was performed to assess the antagonism of nonaflatoxigenic strains against the aflatoxigenic ones. Results revealed that 9% MPA exhibited the highest growth and AFB_1_ inhibition by nonaflatoxigenic strains. It was also found that different nonaflatoxigenic strains exhibited different antagonism against the aflatoxigenic ones which ranged from 11.09 ± 0.65% to 14.06 ± 0.14% for growth inhibition, and 53.97 ± 2.46% to 72.64 ± 4.54% for AFB_1_ inhibition. This variability could be due to the difference in antagonistic metabolites produced by different nonaflatoxigenic strains assessed in the present study. Metabolomics study to ascertain the specific metabolites that conferred the growth and aflatoxin inhibition is ongoing.

## Graphical abstract

The present study suggested that nonproducing sclerotia type *A. flavus* (strain A19R) exhibited effective aflatoxin reduction on A8R on both commercial and semi‐synthetic growth media

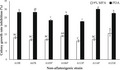

## 1 INTRODUCTION

*Aspergillus* section *Flavi* is considered an opportunistic food spoilage fungal section capable of survival under harsh conditions and might invade a wide range of agricultural products pre‐ and postharvest such as during storage, handling, and shipment (Mamo et al., 2020). These species can survive at a humidity above 80% and temperatures ranging from 12 to 48°C, with the optimum range from 28 to 37°C (Thathana et al., 2017). Under favorable ecophysiological conditions, *Aspergillus* section *Flavi* colonization of food commodities such as beans, cereals, dried fruits, oilseeds, and spices could lead to contamination and accumulation of the mycotoxin aflatoxins (Reddy et al., 2011). *Aspergillus flavus* and *A. parasiticus* are the two most notable aflatoxigenic members of *Aspergillus* section *Flavi*.

Of the 18 different analogs of aflatoxins currently known, aflatoxin B‐series (aflatoxins B_1_ and B_2_; fluoresce blue under UV) and G‐series (aflatoxins G_1_ and G_2_; fluoresce green under UV) are the most frequently found in nature (Benkerroum et al., 2020). Aflatoxins B_1_ and B_2_ are commonly produced by *A. flavus*, while its sister species, *A. parasiticus*, could produce both aflatoxin B‐ and G‐series. The consumption of AFB_1_ from contaminated feedstuff by lactating animals causes it to monohydroxylate into aflatoxin M_1_ (AFM_1_) 12 h after ingestion (Ghaffarian‐Bahraman et al., 2020) and could be secreted into the milk, hence the name M‐series. All three series (B‐, G‐, and M‐series) are significantly important from a food safety point of view as they can cause deleterious effects on human health, and have indirect impacts on the agricultural economy (Norlia et al., 2019). The AFB_1_ has been classified as Group 1 carcinogen by the International Agency for Research on Cancer based on sufficient evidence that demonstrated carcinogenic properties in humans and animals, and its frequent occurrence in foods and feeds worldwide (Caceres et al., 2020).

In nature, corn (*Ze*a *mays* L.) and peanut (*Arachis hypogaea* L.) are the two most commonly contaminated crops by *Aspergillus* section *Flavi*. Contamination of aflatoxins in peanuts by *Aspergillus* section *Flavi* has a negative impact on household food safety, income, and productivity which leads to significant costs and economic losses in developing countries (Waliyar et al., 2015). A lack of awareness regarding the occurrence and risks of aflatoxins, poor agricultural and postharvest practices, and inadequate legislation and regulation have become recurring food safety challenges to combat aflatoxin dietary exposure (Ayelign et al., 2020). The nutritional composition of peanuts such as fats, proteins, fibers, potassium, phosphorous, magnesium, and vitamin B renders them prone to *Aspergillus* section *Flavi* colonization and aflatoxin contamination (Bordin et al., 2014; Tousignant, 2020). *Aspergillus flavus* and *A. parasiticus* are the major biotic stress observed during pre‐ and postharvest stages of peanut cultivation (Jayaprakash et al., 2019). Nevertheless, a study conducted by Dorner & Horn (2007) showed that *A. flavus* was by far the predominant colonizer of peanuts as compared to *A. parasiticus*. Due to this, strict regulations on the acceptable concentration of aflatoxins in peanuts are imposed on the peanut industry worldwide (Tran‐Dinh et al., 1999). In Malaysia, 15 μg/kg of total aflatoxins is set in raw peanuts intended for further processing, and 10 μg/kg in peanut‐based products (Norlia et al., 2018a). In the European Union, a maximum tolerable limit of 2 μg/kg is set for AFB_1_ in foods intended for direct human consumption (European Commission, 2006).

Several approaches have been introduced to overcome the colonization of *A. flavus* and contamination of AFB_1_ in peanuts physically, chemically, or biologically. However, physical control is not often suitable for large scale, and chemical control often leads to environmental pollution. Therefore, biological control offers a safer, environmentally friendly, and economical alternative. Among the biological control approaches, the utilization of nonaflatoxigenic *A. flavus* strains against the aflatoxigenic ones is the most promising (Ehrlich, 2014; Peles et al., 2021). Due to the similar ecophysiological characters that both strains share, their survival within the applied habitat/niche could be guaranteed. This also prevents the introduction of foreign strains and the potential invasiveness that they might bring to the applied habitat/niche. Afla‐Guard and AF36 are two commercial biological control products utilizing the nonaflatoxigenic *A. flavus* strains which have been approved by the U.S. Environmental Protection Agency for the biological control of *A. flavus* and aflatoxin contamination in peanut, corn, and cottonseed with considerable success (Lewis et al., 2019). The local strains may present different responses from the commercial biocontrol, a new study with local isolates would offer a new candidate as potential growth and AFB_1_ inhibitor.

To investigate the growth rate and AFB_1_ production of aflatoxigenic *A. flavus* strains *in vitro*, often the general growth media such as potato dextrose agar (PDA) or malt extract agar (MEA) are used. Since these media provide optimum nutritional components, which will yield luxuriant fungal growth, the risk of under‐ or overestimation of the evaluated parameters prevails. Therefore, it is often best to formulate a semisynthetic fungal growth medium utilizing the crop commodity of interest to better mimic *A. flavus* behavior in nature (Yazid et al., 2018). The optimum concentration of peanuts in the medium will provide better laboratory practice and eliminate the risk of under‐ and overestimation findings. The first objective of the current study is to (i) optimize the suitable concentration of peanuts in a semisynthetic fungal growth medium for the assessment of colony growth rate and AFB_1_ inhibition of aflatoxigenic *A. flavus*. To date, most of the nonaflatoxigenic strains with activity to reduce the colonization of *A. flavus* and production of AFB_1_ are isolated from soil; the strains isolated from food are rarely reported. In this study, seven local nonaflatoxigenic strains isolated from peanuts and peanut product were evaluated for their potential in inhibiting the growth and aflatoxin production of toxigenic *A. flavus*. The second objective is (ii) to evaluate the antagonism potentials of nonaflatoxigenic *A. flavus* local strains isolated from raw peanut kernels against aflatoxigenic *A. flavus* in terms of growth and AFB_1_ inhibition.

## 2 MATERIALS AND METHODS

### 2.1 Chemicals, culture media, and reagents

Potato dextrose agar (PDA) was supplied by Sigma‐Aldrich and was used for the purpose of fungal growth and maintenance. Ingredients involving the preparation of semisynthetic media such as Bacteriological Agar (Sigma‐Aldrich), Chloramphenicol (Fisher Scientific), 1 M HCl (Merck), and 1 M NaOH (Merck) were used. Mix aflatoxin standard (AFB_1_, AFB_2_, AFG_1,_ and AFG_2_) was supplied by Sigma‐Aldrich. Methanol and acetonitrile with HPLC grade were used for extraction and detection of aflatoxin, which were supplied by Merck. Peanut kernels of food grade were purchased from the local market.

### 2.2 Nonaflatoxigenic and toxigenic *Aspergillus flavus* strains

Toxigenic *A. flavus* (A8R) and nonaflatoxigenic *A. flavus* (A19R, A67R, A105P, A106P, A113P, A114P, and A121R) strains obtained from the Laboratory of Food Safety and Food Integrity, Selangor, Malaysia, were used in dual culture assays. The capital letter “A” represents the strain species, which is all *Aspergillus flavus*, and the capital letter at the end of the strain labeling system represents the source of isolated strains, where “R” was isolated from raw peanut kernels and “P” was isolated from various peanut products. Table 1 exhibits the chemotype and morphotype of all the strains used in this study, all strains had been chemically and molecularly identified as *A. flavus* from the previous study (Mahror et al., 2019). The strains were maintained on PDA and stored at 4°C.

**TABLE 1:** Chemotype and morphotype of aflatoxigenic and nonaflatoxigenic strains

Strain	Chemotype	Chemical profile				Sclerotium morphotype		
		AFB	AFG	CPA	Aspergillic acid	L‐type	S‐type	None
A8	I	*		*	*			*
A19R	IV				*			*
A67R	IV				*		*	
A105P	IV				*	*		
A106P	IV				*	*		
A113P	IV				*	*		
A114P	IV				*	*		
A121R	VI				*	*		

*Adapted from* Norlia et al. (2018a).

•: present.

AFB (B1 + B2).

AFG (G1 + G2).

CPA (cyclopiazonic acid).

L‐type sclerotium (>400 μm in diameter).

S‐type sclerotium (<400 μm in diameter).

None: Did not produce sclerotium.

### 2.3 Preparation of spore suspension

The spore suspension was prepared by pouring 1000 μl of sterile distilled water containing 0.05% Tween 80 on top of a hyphal mat of 7 dayS culture on a PDA plate, and the spores were retrieved by gently shaking the culture plate. The spore suspensions were transferred into a sterile 2 ml centrifuge tube, and the initial spore concentration was calculated using a hemocytometer and diluted until the concentration reach 1 × 10^6^ spore/ml (Olagunju & Ijabadeniyi, 2020). The spore suspension was stored at 4°C for further analysis.

### 2.4 Preparation of PDA and MPA

PDA was prepared according to the guideline from the manufacturers and autoclaved at 121°C for 15 min. MPA was prepared by following by method described by Yazid et al. (2018). Peanut kernels were oven‐dried for 24 h and ground using a laboratory blender. Then, different amounts of ground peanuts were mixed together with 1 l of distilled water to achieve the desired concentration of MPA (1,3, 5, 9, 10, 20, and 30%). Fifteen grams of Bacteriological Agar (Sigma‐Aldrich) and 10 mg chloramphenicol were added to the peanut suspension and vigorously shook until the ingredient had completely dissolved. The pH of the peanut suspension was adjusted with either 1 M HCl or 1 M NaOH until the pH value reached 5.6, after which the suspension was sterilized by autoclaving the suspension at 121°C for 15 min. Approximately 20 ml of media per plate was poured, and the media remained at room temperature until the solidification had been completed.

### 2.5 Dual culture assay

A dual culture assay was performed according to Toyotome et al. (2019) with some modifications. Approximately 20 μl of toxigenic *A. flavus* (A8R) spore suspension (1 × 10^6^ spore/mL) was loaded and inoculated at 20 mm from the edge of a Petri plate (Ø 90 mm). On the opposite side, 20 μl of the spore suspension of nonaflatoxigenic *A. flavus* strains was inoculated. Plate inoculated with only toxigenic strains serves as a negative control. All plates were incubated at 30 ± 5 for 7 days. Control and treatments were carried out in triplicate.

The colony growth rate of toxigenic *A. flavus* strains was obtained by using a formula (Yazid et al., 2018). The hyphal colony expansion was recorded every 24 h by measuring the colony diameter in two directions perpendicularly from the point of inoculation, and the average two‐directional diameter was calculated and expressed as the final diameter of the colony. The growth rate was obtained by plotting *y* = *mx* + *c*, where *y* was the average of the colony diameter (mm), *m* was the growth rate (mm/day), *x* was the incubation period (d), and C was set to 0 as there is no hyphal formation at the beginning of inoculation. The percentage colony growth rate inhibition (%) was obtained as prescribed by Gong et al. (2014) with slight modification using the following formula:

$$\text{inhibition}=\frac{\left(G^{{}^{\circ}}-G1\right)}{G^{{}^{\circ}}}\times 100\%$$

where G° is the colony growth rate of the control plate (mm/day) and G^1^ is the colony growth rate (mm/day) of the cocultured plate.

### 2.6 Extraction of AFB_1_

The extraction of AFB_1_ from the toxigenic strains was performed based on the method described by Norlia et al. (2018b). In the dual culture plate, three agar plugs (Ø 6 mm) from the toxigenic strain colony were collected at 5 mm away from the edge of nonaflatoxigenic strains, while three agar plugs were randomly collected from the control plate. The agar plugs were transferred into a 2 ml microcentrifuge tube and a total weight of the agar plugs was obtained.

Then, 1 ml of methanol was added into the microcentrifuge tube containing agar plugs, and the mixture was left at room temperature for 60 min. In the meantime, the mixture was vortexed three times for 1 min. After that, the mixture was centrifuged at 4000 rpm for 30 min. The mixture was filtered using a syringe nylon filter (0.45 μm) and transferred into amber HPLC vials.

### 2.7 Detection of AFB_1_ by HPLC

Detection of AFB_1_ in hyphal plugs extract was performed using the method described by Norlia et al. (2018a) with a slight modification. The reverse‐phased Symmetry C18 column (Waters,) with 5 μm particle size and dimension of 250 mm × 4.6 mm was attached to a HPLC system (Waters 600,) to separate AFB_1_ from other components in the extract. The C18 column was connected to the postcolumn Photochemical Reactor For Enhanced Detection (PHRED; Aura Industries,). The extract was exposed to UV light in the postcolumn and enhanced the natural fluorescence properties of AFB_1_. At the end of HPLC system, a fluorescence detector (Waters, 2475) was installed to detect the presence of AFB_1_ in the enhanced extract. The wavelength was set at 365 nm for excitation and 435 nm for emission of AFB_1_. Twenty μL of hyphal plugs extract was injected into the system and run with a mobile phase composition of methanol/deionized water/acetonitrile (35:55:10, v/v/v) at the flow rate of 0.6 ml/min. The injection was carried out in triplicate. Data processing, acquisition, and reporting were carried out by Empower 2 Chromatography software (Waters,). Each solvent in the mobile phase was filtered separately using a 0.45 μm filter membrane. The percentage AFB_1_ inhibition (%) was obtained as described by Gong et al. (2014) with slight modification by using the following formula:

$$\text{inhibition}=\frac{\left(B^{{}^{\circ}}-B1\right)}{B^{{}^{\circ}}}\times 100\%$$

where B° is the concentration AFB_1_ of the control plate (ng/g) and B^1^ is the concentration AFB_1_ (ng/g) of the cocultured plate.

The percentage of AFB_1_ inhibition was evaluated by comparing the AFB_1_ concentration of toxigenic strains in the control plate with the AFB_1_ concentration of toxigenic strains in the cocultured plate.

### 2.8 Method validation

The calibration curve was constructed by injecting seven different concentrations of AFB_1_ (1, 5, 10, 50, 100, 500, and 1000 ng/ml); each of the concentrations was injected three times (∑ = 21) intermittently throughout the sample analysis to confirm the repeatability and reproducibility (Yazid et al., 2018). The regression coefficient (*R* ^*2*^) for the calibration curve was 0.9965. A linear regression model was used to obtain the limit of detection (LOD) and limit of quantification (LOQ) of the present analysis by using the formula: LOD = 3Sa/b and LOQ = 10Sa/b, where Sa is the standard deviation of the response and b is the slope of the calibration curve. The LOD and LOQ obtained from this analysis were 1.75 ng/g and 5.79 ng/g, respectively. The chromatogram peak of AFB_1_ was observed at 30.18 ± 0.5 min; any peak appearing ±0.51 min was rejected.

### 2.9 Optimization of MPA

The suitable concentration of peanut in MPA was determined by dual culturing aflatoxigenic strains (A8R) with selected nonaflatoxigenic strain (A121R); the selection of nonaflatoxigenic strains were involved in the preliminary study. Peanut concentration showed a significant colony growth rate and aflatoxin B_1_ inhibition was selected and used in the further experiment.

### 2.10 Antagonism potentials of nonaflatoxigenic strains

The antagonism potential of nonaflatoxigenic strains (A19R, A67R, A105P, A106P, A113P, A114P, and A121R) was determined by evaluating the colony growth rate and aflatoxin B_1_ inhibition of toxigenic strain (A8). Nonaflatoxigenic strains that showed significant inhibition were selected as most promising as an antagonism strain/s.

### 2.11 Statistical analysis

All the analyses were conducted in triplicate per analysis and statistical analysis was performed by using Minitab® Version 16 for Windows (Minitab Inc.). The data were expressed as a mean ± standard error. The statistical analysis was performed using analysis on variance (ANOVA) in order to determine the significant difference by Fisher's least significant test with mean separation (*p* < .05).

## 3 RESULT

### 3.1 Visual observation of aflatoxigenic *A. flavus* (strain A8R) cocultivated with nonaflatoxigenic *A. flavus* (strain A121R) on different types of growth media

**FIGURE 1:**
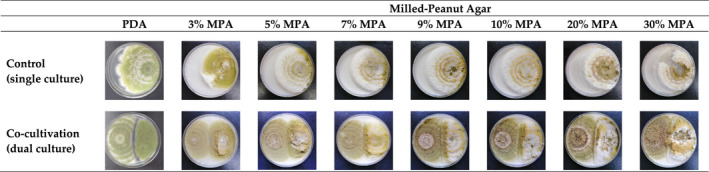
Hyphal expansion of aflatoxigenic *Aspergillus flavus* (strain A8R; right) cocultivated with nonaflatoxigenic *A. flavus* (strain A121R; left) on 90 mm Ø potato dextrose agar (PDA) and milled‐peanut agar (MPA) plates at different peanut concentrations (3, 5, 7, 9, 10, 20, and 30% w/v). Plates were incubated at 30°C for 7 days, and hyphal expansion was recorded daily

Figure 1 shows the hyphal expansion of aflatoxigenic *A. flavus* (strain A8R) cultivated individually and cocultivated with nonaflatoxigenic *A. flavus* (strain A121R), on PDA and MPA at different peanut concentrations after 7 days of incubation at 30°C. Following individual cultivation, the colony of aflatoxigenic *A. flavus* on PDA grew radially from the point of inoculation, while exhibiting the characteristic yellowish‐green color of conidia. Similar macromorphology was also observed on MPA except that the colonies on MPA were thicker than that on PDA. However, it was noted that as the concentration of peanut increased, the thickness of the mycelial mat also increased, and velvet‐like texture became more apparent, while yellowish‐green conidia became lesser and restricted only around the inoculation point. On the cocultivated PDA plate, both aflatoxigenic and nonaflatoxigenic colonies stopped expanding outward just before coming into contact with each other thus exhibiting a clear “antagonistic gap,” with the aflatoxigenic colony showing a slightly smaller colony size. On cocultivated MPA plates, a similar trend of nonaflatoxigenic outgrowing aflatoxigenic was observed and became more apparent as the peanut concentration increased. Nonaflatoxigenic colonies also had vigorous conidiation as compared to aflatoxigenic regardless of peanut concentration. Visually, the 9% MPA plate and the plates with higher peanut concentration onward exhibited the darkest green conidiation, but with no apparent difference between them.

### 3.2 Colony growth rate inhibition of aflatoxigenic *A. flavus* (strain A8R) cocultivated with nonaflatoxigenic *A. flavus* (strain A121R) on different types of growth media

**FIGURE 2:**
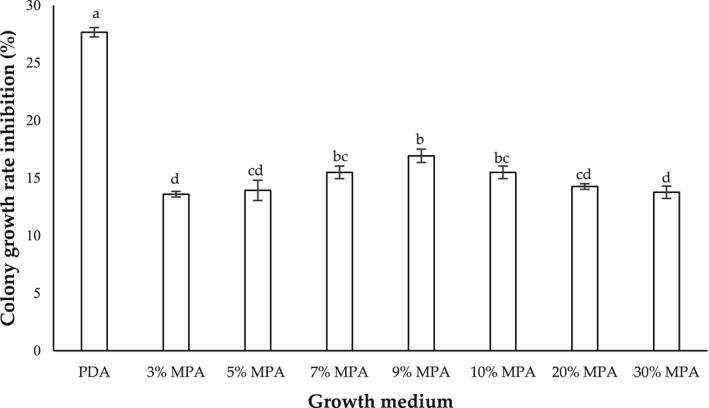
Colony growth rate inhibition (%) of aflatoxigenic *Aspergillus flavus* (strain A8R) cocultivated with nonaflatoxigenic *A. flavus* (strain A121R) on potato dextrose agar (PDA) and milled‐peanut agar (MPA) plates at different peanut concentrations (3, 5, 7, 9, 10, 20, 30% w/v), and incubated at 30°C for 7 days. Data are means of triplicate (*n* = 3) with bars indicating a standard error (SE). Small letters indicate a significant difference (*p* < .05) using least significant difference (LSD)

Figure 2 shows the colony growth rate inhibition (%) of aflatoxigenic *A. flavus* (strain A8R) cocultivated with nonaflatoxigenic *A. flavus* (A121R) on PDA and MPA at different peanut concentrations and incubated at 30°C for 7 days. Strain A121R exhibited the highest inhibition percentage (27.67 ± 0.40%) against strain A8R on PDA as compared to on MPA. On MPA, strain A121R inhibited strain A8R from 13.60 ± 0.25% to 16.93 ± 0.58%, where 7, 9 and 10% MPA yielded the highest inhibition percentage, with 9% being the highest average inhibition percentage.

### 3.3 AFB_1_ inhibition of aflatoxigenic *A. flavus* (strain A8R) cocultivated with nonaflatoxigenic *A. flavus* (strain A121R) on different types of growth media

**FIGURE 3:**
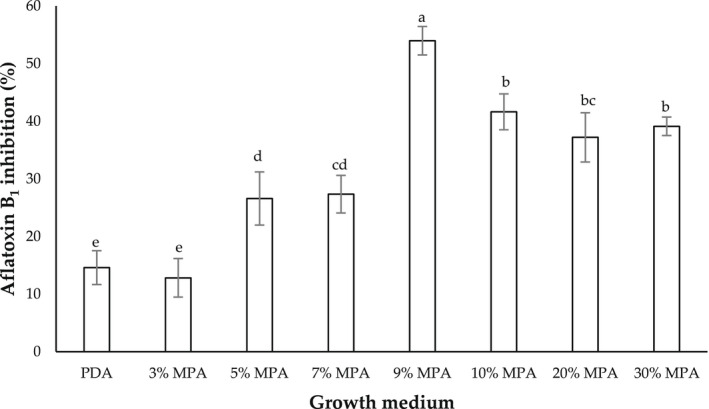
Aflatoxin B1 inhibition (%) of aflatoxigenic *Aspergillus flavus* (strain A8R) cocultivated with nonaflatoxigenic *A. flavus* (strain A121R) on potato dextrose agar (PDA) and milled‐peanut agar (MPA) plates at different peanut concentrations (3, 5, 7, 9, 10, 20, and 30% w/v), and incubated at 30°C for 7 days. Data are means of triplicate (*n* = 3) with bars indicating a standard error (SE). Small letters indicate a significant difference (*p* < .05) using least significant difference (LSD)

Figure 3 shows the AFB_1_ inhibition (%) of aflatoxigenic *A. flavus* (strain A8R) cocultivated with nonaflatoxigenic *A. flavus* (A121R) on PDA and MPA at different peanut concentrations and incubated at 30°C for 7 days. As opposed to growth inhibition (Figure 1), strain A121R exhibited the highest inhibition percentage (53.97 ± 2.57%) against strain A8R on 9% MPA as compared to the other tested peanut concentrations, as well as on PDA (control). Since 9% MPA exhibited the highest inhibition against both growth rate and AFB_1_ production of strain A8R, it was selected as the growth medium for subsequent study.

### 3.4 Visual observation of aflatoxigenic *A. flavus* (strain A8R) cocultivated with different nonaflatoxigenic *A. flavus* strains on 9% MPA and PDA

**FIGURE 4:**
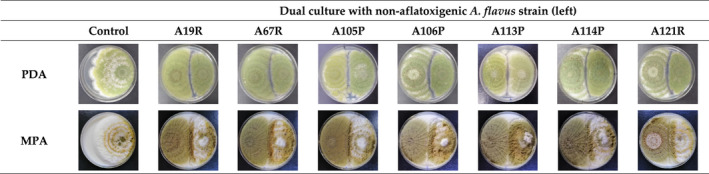
Hyphal expansion of aflatoxigenic *Aspergillus flavus* (strain A8R; right) cocultivated with different nonaflatoxigenic *A. flavus* strains (left) on 90 mm Ø 9% milled‐peanut agar (9% MPA) plates. Plates were incubated at 30°C for 7 days, and hyphal expansion was recorded daily

Figure 4 shows the hyphal expansion of aflatoxigenic *A. flavus* (strain A8R) cocultivated with various nonaflatoxigenic *A. flavus* strains on PDA and 9% MPA after 7 days of incubation at 30°C. Similar macromorphology as shown in Figure 1 is also shown in Figure 4. On PDA, all *A. flavus* cultures exhibited yellowish‐green conidia and less thick hyphal mats, whereas on 9% MPA, the conidia were much darker, and the hyphal mats were velvety. Between different nonaflatoxigenic strains, different antagonism was exhibited on both PDA and 9% MPA. However, on 9% MPA, the antagonism was observed at a higher degree. For example, on PDA, all dual cultures exhibited a clear “antagonistic gap,” whereas, on 9% MPA, this was not clearly visible.

### 3.5 Colony growth rate inhibition of aflatoxigenic *A. flavus* (strain A8R) cocultivated with different nonaflatoxigenic *A. flavus* strains on 9% MPA and PDA

**FIGURE 5:**
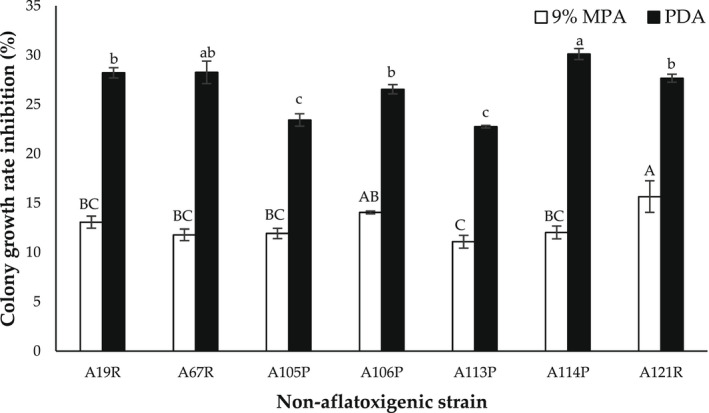
Colony growth rate inhibition (%) of aflatoxigenic *Aspergillus flavus* (strain A8R) cocultured with different nonaflatoxigenic *A. flavus* strains on potato dextrose agar (PDA) and 9% milled‐peanut agar (9% MPA) plates, incubated at 30°C for 7 days. Data are means of triplicate (*n* = 3) with bars indicating a standard error (SE). Capital letters indicate a significant difference (*p* < .05) between 9% MPA plates using least significant difference (LSD). Small letters indicate a significant difference (*p* < .05) between PDA plates using least significant difference (LSD)

Figure 5 shows the colony growth rate inhibition (%) of aflatoxigenic *A. flavus* (strain A8R) cocultivated with different nonaflatoxigenic *A. flavus* strains on 9% MPA and PDA plates and incubated at 30°C for 7 days. The percentage of colony growth rate inhibition of all nonaflatoxigenic strains was higher on PDA (22.75 ± 0.12%–30.12 ± 0.55%) as compared to on 9% MPA (11.09 ± 0.65%–14.06 ± 0.14%). Nonaflatoxigenic strain A114P A67R yielded the highest colony growth rate inhibition, with A114P exhibiting the highest average colony growth inhibition (30.12 ± 0.55%) on PDA. However, for nonaflatoxigenic strain, A121R and A106P yielded the highest inhibition, with A121R exhibiting the highest average colony growth inhibition (14.06 ± 0.14%) on 9% MPA.

### 3.6 AFB_1_ inhibition of aflatoxigenic *A. flavus* (strain A8R) cocultivated with different nonaflatoxigenic *A. flavus* strains on 9% MPA and PDA

**FIGURE 6:**
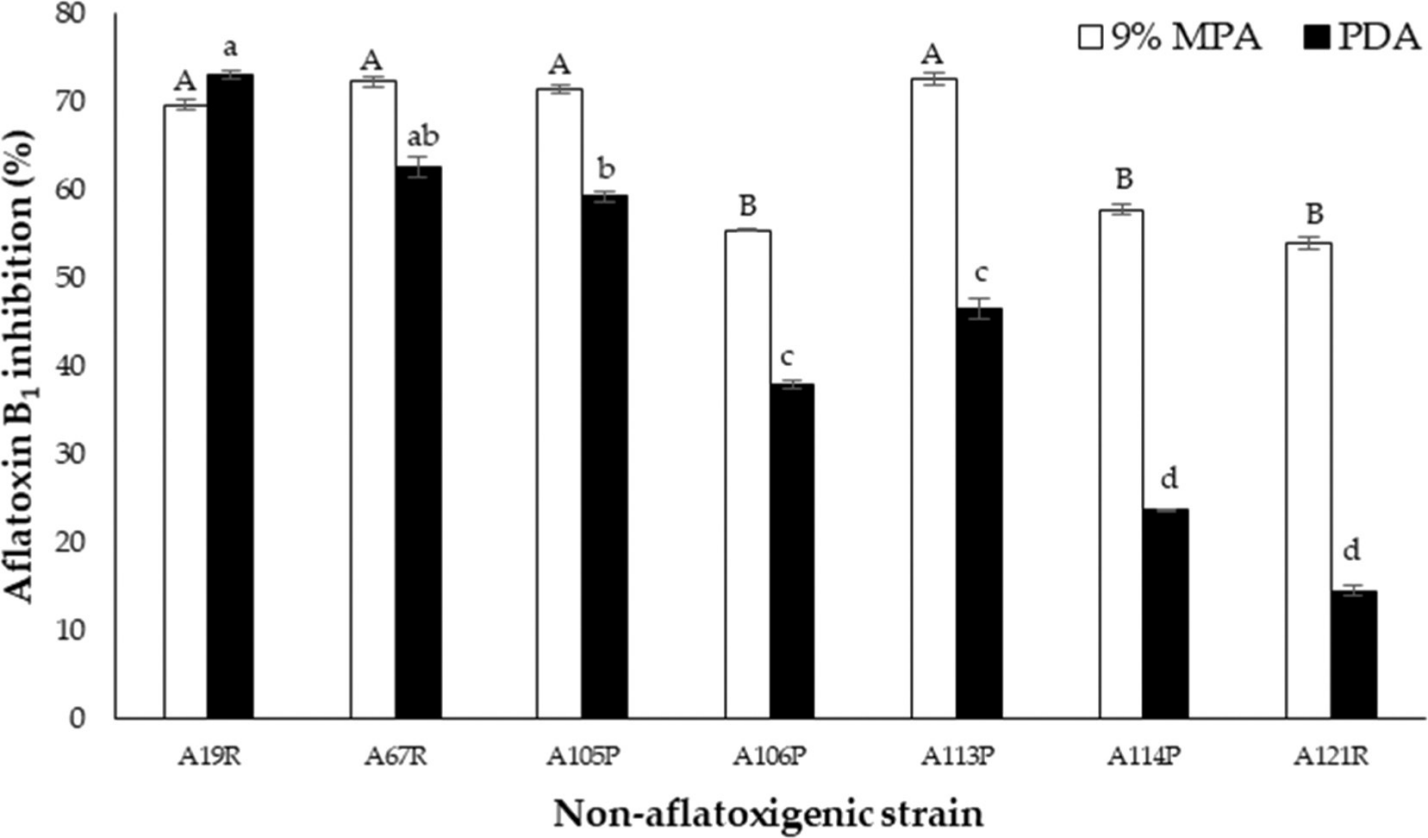
Aflatoxin B_1_ inhibition (%) of aflatoxigenic *Aspergillus flavus* (strain A8R) cocultured with different nonaflatoxigenic *A. flavus* strains on potato dextrose agar (PDA) and 9% milled‐peanut agar (9% MPA) plates, incubated at 30°C for 7 days. Data are means of triplicate (*n* = 3) with bars indicating a standard error (SE). Capital letters indicate a significant difference (*p* < .05) between 9% MPA plates using least significant difference (LSD). Small letters indicate a significant difference (*p* < .05) between PDA plates using least significant difference (LSD)

Figure 6 shows the AFB_1_ inhibition (%) of aflatoxigenic *A. flavus* (strain A8R) cocultivated with different nonaflatoxigenic *A. flavus* strains on 9% MPA and PDA plates and incubated at 30°C for 7 days. On 9% MPA, all nonaflatoxigenic strains inhibited the AFB_1_ production of strain A8R above 50% (53.97 ± 2.46%–72.64 ± 4.54%). On PDA, the AFB_1_ inhibition widely varied (14.60 ± 2.94% by strain A121R to 73.15 ± 2.01% by strain A19R). Strain A19R yielded the highest inhibition on both 9% MPA and PDA, at 69.72 ± 2.00% and 73.15 ± 2.01%, respectively, while strain A121R yielded the lowest on both 9% MPA and PDA, at 53.97 ± 2.46% and 14.60 ± 2.94%, respectively.

### 3.7 Colony growth rate and AFB_1_ production inhibition of different morphotype nonaflatoxigenic *A. flavus* strains against toxigenic strain

Table 1 shows the list of morphotypes of toxigenic and nonaflatoxigenic strains used in this study; the toxigenic strain A8R belongs to the nonproducer. The colony growth rate and AFB_1_ inhibition activity varied among the morphotypes of nonaflatoxigenic strains. Strains A121R that belongs to the L‐type sclerotia group exhibited the highest colony growth rate inhibition (14.06 ± 0.14%) on 9% MPA. For AFB_1_ inhibition, the highest was exhibited by the nonproducer strain (A19R) (69.72 ± 2.00%).

## 4 DISCUSSION AND CONCLUSION

The present study aimed to investigate the potential antagonism of several nonaflatoxigenic *A. flavus* strains isolated from peanuts against one aflatoxigenic strain *in vitro* using the in‐house formulated semisynthetic peanut‐based growth medium (Milled‐Peanut Agar; MPA) in terms of colony growth rate and AFB_1_ inhibition. The growth medium was formulated and used to simulate the natural crop commodity often colonized by *Aspergillus* section *Flavi* and reduce the risk of under‐ or overestimation of the evaluated parameters when using general growth media such as potato dextrose agar (PDA) or malt extract agar (MEA).

To formulate the semisynthetic growth medium, the peanut concentration must first be optimized, in the sense that it would support good hyphal expansion and aflatoxin production of the pathogen in question. Based on Figure 1, 9% MPA was observed to visually support good fungal growth and was not significantly different from 10, 20, or 30% peanut concentration. Therefore, 9% MPA was selected as it required the lowest amount of milled‐peanut powder, as compared to 10%, 20%, or 30% peanut concentration. Initially, 40% and 50% peanut concentrations were also prepared into growth media, but they resulted in lumpy agar texture and inconsistent hyphal expansion (due to the lumpiness, which hindered the calculation of colony growth rates); thus, these concentrations were excluded from the present study. However, visualization is just a qualitative measure. To further ascertain the optimum peanut concentration, quantitative measurements were conducted by quantifying the growth rate (which is reported in growth rate inhibition percentage; Figure 2), and AFB_1_ production (which is reported in AFB_1_ inhibition percentage; Figure 3) on the different peanut concentrations. The inhibition data were derived from the difference in those parameters when aflatoxigenic strain A8R was grown in single culture (control) and in dual culture (cocultivation with nonaflatoxigenic strain 121R). A dual culture assay is one of the standard methods to assess the performance of two antagonizing microbial strains (Rahman et al., 2009). It is crucial to also assess the growth performance of the potential antagonist strain (nonaflatoxigenic strain 121R) because before it could antagonize the aflatoxigenic strain, it must first be able to grow on the synthesized growth medium. As expected, based on Figures 2 and 3, 9% MPA yielded the highest average growth rate and AFB_1_ inhibition percentages as compared to the other tested peanut concentrations. This parallels the qualitative visualization presented in Figure 1. As the colony growth rate of nonaflatoxigenic increases, it will be able to surpass the colony growth rate of toxigenic strains and lead to greater colony growth rate inhibition. High colony growth rate inhibition was able observed in PDA compared to MPA; this observation might be due to the different nutrient compositions in both growth mediums used in this study. A nutrient composition (simple sugar, protein, and micronutrients) is one of the biotic factors that influence the rate of colonization and secondary metabolites production of *A. flavus*. Potato dextrose agar is made up of 4 mg of potato extract and 20 mg of simple sugar (dextrose); from the nutritional fact of potato (Raidl, 2020), it contains 26 g of carbohydrate, 3 g of protein, and 0 g of fats per serving. Peanut contains 4.6 g of carbohydrate, 7.3 g of protein, and 14 g of fats per serving. The study conducted by Liu et al. (2016) has reported that the increment of starch into growth media enhances the colony growth rate of *A. flavus*, with the presence of a high source of starch in PDA, it will supply a luxuriant environment for *A. flavus* to grow in the medium. Liu et al. (2016) also reported that the removal of lipids from medium substrates significantly reduced the potential for AFB_1_ production by *A. flavus*. As MPA contains high lipid and protein, more AFB_1_ was produced in MPA compared to PDA, which was observed before the treatment with the nonaflatoxigenic strains (in a control plate). With low production of AFB_1_ observed in PDA, a small number of AFB_1_ was suppressed by the nonaflatoxigenic strains leading to low AFB_1_ inhibition in PDA compare to MPA. Ecophysiologically, fungal growth and mycotoxin production are often not parallel (Daou et al., 2021), as evidenced in Figures 2 and 3. Among all the concentrations of MPA, 9% of peanut concentration in the medium exhibits high inhibition on both colony growth rate and AFB_1_ inhibition, which was used in subsequent experiments.

In terms of macromorphology on PDA, the visualization of aflatoxigenic *A. flavus* (strain A8R) is in line with previous studies. A white border of sporulating mycelia of toxigenic *A. flavus* strains isolated from peanuts in Kenya was observed on PDA at 28°C. The colony then continued to sporulate and produce more conidia, and the colony became slightly raised as mycelia piled and the center became floccose and rough. After 10 days, almost the entire colony turned green with white mycelia encircling the colony (Okayo et al., 2020). Findings reported in Yazid et al. (2018) are also in line with the present study, where the incubation of aflatoxigenic *A. flavus* NRRL 3357 on PDA at various temperatures (30°C suggested to be the optimum temperature) yielded a tremendous amount of green conidia on the velutinous and floccose hyphal mat after 7 days incubation. Currently, however, there is no macroscopic visualization of nonaflatoxigenic *A. flavus* strains reported on a semisynthetic peanut‐based growth medium.

To further ascertain the potential antagonism of nonaflatoxigenic strain A121R, different nonaflatoxigenic strains were cocultivated with aflatoxigenic strain A8R on 9% MPA in the second part of the present study. Nonaflatoxigenic strain A121R yielded the highest average colony growth rate inhibition among the tested nonaflatoxigenic strains (Figure 5). On AFB_1_ inhibition, however, two clusters appeared. Cluster 1 (69.72%–72.65%) was exhibited by nonaflatoxigenic strains A19R, A67R, A105P, and A113P, while the slightly lower Cluster 2 (55.50%–57.87%) was dominated by nonaflatoxigenic strains A106P, A114P, and A121R. The variability in growth and aflatoxin antagonism might be correlated with genetic differences (cutinase gene and secondary metabolite gene cluster) as a result of different morphotypes of nonaflatoxigenic strains used.

The morphologies of *A. flavus* strains can be grouped into two types: S morphotype (S‐type) which produces many small sclerotia but few conidia, and L morphotype (L‐type) which produces fewer large sclerotia but many conidia. Strains that do not produce any sclerotia in any conditions were considered nonproducer strains (Pildain et al., 2004; Ohkura et al., 2018). In the present study, the nonaflatoxigenic strains belonged to one S‐type (A67R), five L‐type (A105P, A106P, A113P, A114P, A121R), and one nonproducer (A19R) strains, and grouped into chemotype IV, which means that the strains do not produce aflatoxins or cyclopiazonic acid. The aflatoxigenic strain A8R belonged to the nonproducer, and was grouped in Chemotype I, as the strain produced B‐type aflatoxins and cyclopiazonic acid. The chemotype and morphotype of strains used in the present study have been reported in our previous works (Norlia et al., 2018a; Norlia et al., 2018b).

Results obtained from the present study suggest that nonproducing sclerotia type *A. flavus* (strain A19R) exhibited effective aflatoxin reduction on A8R on both commercial and semisynthetic growth media. The study conducted by Chang et al. (2005) mentioned that nonaflatoxigenic *A. flavus* strain was more effective in inhibiting the production of AFB_1_ of a similar morphotype of the aflatoxigenic *A. flavus* strain. Similarly, nonaflatoxigenic L‐type strain (TX9‐8) *A. flavus* isolated from the cotton field was found more effective to control the production of AFB_1_ from aflatoxigenic L‐type *A. flavus* as compared to S‐type *A. flavus* strains (Chang et al., 2006; Hua et al., 2019). Another study conducted by Cotty (1997) reported the potential of aflatoxin reduction between similar and different morphotypes of aflatoxigenic and nonaflatoxigenic *A. flavus* strains. In terms of percentage inhibition, nonaflatoxigenic strain CA12 (L‐type) reduced AFB_1_ by 60%–70% from both morphotypes of aflatoxigenic strains, while AF123 (also L‐type) only reduced AFB_1_ by 20%–40% from both morphotypes (Chang et al., 2015). This shows that even among similar morphotypes, the percentage of inhibition would differ. In the present study, different morphotypes of nonaflatoxigenic strains used (nonproducer, L‐type, S‐type) reduced AFB_1_ by 50%–75% against aflatoxigenic strain A8R (nonproducer) on 9% MPA. A comparative genomic study between two morphotypes of aflatoxigenic *A. flavus* strains conducted by Ohkura et al. (2018) suggested that genetic differences might play a role in the variable adaptation to the environment as they adapted to their different niches.

This study aimed at optimizing the suitable concentration of peanut in a semisynthetic growth medium for the assessment of colony growth rate and AFB_1_ inhibition, which were further used for evaluating the antagonism potentials of nonaflatoxigenic *A. flavus* against aflatoxigenic *A. flavus* in terms of growth and AFB_1_ inhibition. At 9%, peanut concentration exhibits the highest average colony growth rate and AFB_1_ inhibition; this finding reduces the potential of the risk of under‐ and overestimation findings in the *in vitro* assessment. Two candidates from all tested nonaflatoxigenic strains were short‐listed with potential colony growth and AFB_1_ reduction. Strain A121R was suggested as a potential colony growth rate inhibitor while A19R was a potential AFB_1_ inhibitor.

## FUNDING INFORMATION

The present study was financially supported by UPM through the *Geran Putra Berimpak* (GBP) grant scheme (UPM/800–3/3/1/GPB/2018/9658100).

## CONFLICT OF INTEREST

The authors declare no conflict of interest.
